# Immobilized *Lacticaseibacillus paracasei* on Sunflower Seeds as a Stable Functional Ingredient for Cream Cheese

**DOI:** 10.3390/microorganisms14030671

**Published:** 2026-03-16

**Authors:** Chrysoula Pavlatou, Anastasios Nikolaou, Yiannis Kourkoutas

**Affiliations:** Laboratory of Applied Microbiology and Biotechnology, Department of Molecular Biology & Genetics, Democritus University of Thrace, 68100 Alexandroupolis, Greece; xrysapavl@gmail.com (C.P.); anikol@mbg.duth.gr (A.N.)

**Keywords:** functional foods, probiotics, immobilization, wild-type strain, cream cheese

## Abstract

During the last few decades, an urgent need for sustainable and health-promoting food products has been witnessed. In this vein, the development of functional foods enriched with probiotics has gained considerable interest from both the food industry and consumers. However, the maintenance of high cell viability until the time of consumption remains a significant challenge. In this study, freeze-dried immobilized *Lacticaseibaciilus paracasei* FBM_1327 cells on sunflower seeds were evaluated as a functional food ingredient, and their ability to survive during simulated digestion and storage at ambient and refrigerated temperatures in comparison to free cells was assessed. Cell immobilization resulted in higher survival rates (>70%) after *in vitro* digestion compared to free cells (<40%), while the freeze-dried immobilized cells maintained in cell levels >7.5 log cfu/g during storage for 6 months at 4 °C. In the next step, freeze-dried free or immobilized cells were incorporated in cream cheese (CCF and CCI samples, respectively) at a concentration of >8 log cfu/g. Cell viability of the immobilized cells remained stable (>8.1 log cfu/g) during storage, while live cell counts of free cells dropped to 7.51 ± 0.11 log cfu/g after 28 days. The fortification of cream cheese with immobilized *L. paracasei* FBM_1327 cells on sunflower seeds improved the volatile compounds profile, while all samples were accepted by the panel during the sensory evaluation.

## 1. Introduction

The development of novel foods associated with health benefits has attracted the attention of both the food industry and consumers today. The term “functional foods” refers to food enriched with vitamins, bioactive compounds, minerals, prebiotics, or probiotics that could confer a positive effect on consumers’ health [1].

Probiotic-enriched foods play a central role in the functional foods sector, particularly in supporting gut health. According to FAO/WHO, “probiotics are live microorganisms that when administered in adequate amounts can confer a health benefit on the host”. Hence, probiotics are linked with beneficial health effects, including antagonistic activity against food-borne pathogens and protection from diseases like diabetes, hypercholesterolemia, inflammatory bowel disease, and others [2,3,4], while they modulate and balance the composition of the intestinal microbiome [5,6]. Although there is no specific dosage for all probiotics, a minimum recommended concentration of 7 log cfu/g at the time of consumption should be ensured in order to confer their beneficial properties [7].

The effectiveness of probiotic microorganisms is strongly affected by cell viability after administration. During gastrointestinal tract (GI) transit, probiotics should be able to survive the presence of digestive enzymes, acids, and bile salts in order to proliferate and colonize the intestinal mucosa and exert the beneficial properties. Cell viability is also influenced by the food processing chain (production, packaging, and storage). To overcome these obstacles, cell immobilization is considered a promising approach [8]. Immobilization can be defined as the natural ability of cells to adhere to surfaces or to become entrapped within a specific porous matrix [8]. By forming a protective physical barrier and a stabilizing microenvironment, immobilized cells exhibit enhanced resistance to environmental stress. According to the literature, immobilized cells exhibit higher viability under various storage temperatures compared to free cells [9], while immobilization offers protection during passage through the harsh conditions present in the GI tract [10]. In addition, the incorporation of immobilized cells into foods may lead to improved organoleptic characteristics and enhanced resistance to microbial spoilage, resulting in extended shelf life in some cases [11,12].

Natural food ingredients, such as fruit pieces, cereals, or nuts, have been evaluated as suitable food carriers [8,9,13,14]. In particular, immobilization of probiotics on food ingredients with prebiotic potential may lead to the development of functional symbiotic foods. Sunflower seeds (*Helianthus annuus*) are rich in unsaturated fatty acids and bioactive compounds, including phenolic acids and tocopherols that have been associated with antioxidant and anti-inflammatory properties [15]. Furthermore, their rich content of dietary fibers has also been linked to beneficial modulation of gut microbiota composition, indicating a prebiotic potential [16]. Owing to these functional attributes, sunflower seeds may serve as a promising vehicle for probiotic delivery.

Τhe selection of a suitable food matrix is crucial for achieving high cell concentrations in a food product. Although there is a wide range of foods enriched with probiotic cultures in the market worldwide, such as cereals, juices, and chocolates, dairy products represent the most common vehicle for probiotics [17]. As platforms for the delivery of probiotics, dairy products are not only traditionally accepted by consumers, but their storage at low temperatures and short shelf life favor the maintenance of high cell viability [18,19]. Among them, cheeses, particularly cream cheese, are considered suitable substrates due to their relatively high pH, fat content, water activity, and solid structure. These properties help buffer the cells during GI transit, thereby enhancing their survival [20].

The rapid expansion of the probiotic industry in recent years has increased the demand for novel wild-type probiotic strains. In this vein, selected strains of *Lactobacillus* spp. have been widely studied not only for their probiotic potential, but also for their functional and technological characteristics. Notably, certain *Lacticaseibacillus paracasei* strains have been tested as starter or adjunct cultures in cheese production, and their incorporation has been associated with the enhancement of the sensory profile of the products, mainly through cells’ metabolic activity [21,22,23]. Hence, the wild-type strain *L. paracasei* FBM_1327, isolated from goat milk, has been previously evaluated for its probiotic potential *in vitro* by our research group [11] and exhibited strong cholesterol assimilation potential, antagonistic activity against pathogens, and high survival rates in acidic and osmotic environments. Moreover, the incorporation of immobilized *L. paracasei* FBM_1327 cells on oat flakes into yogurt and ayran products had no adverse effect on the physicochemical and sensory attributes, while cell loads remained >7 log cfu/g during storage [11].

In this context, freeze-dried *L. paracasei* FBM_1327 cells on sunflower seeds were initially evaluated as a stable functional food ingredient, and the development of cream cheese enriched with the functional food ingredient was followed. In the present study, data on the effect of *L. paracasei* FBM_1327 cells on their survival and on the physicochemical properties of cream cheese during storage are presented.

## 2. Materials and Methods

### 2.1. Microbial Strain

*L. paracasei* FBM_1327 was cultured in a synthetic food-grade medium developed by our research group (unpublished data) with the following composition: Glucose (20 g/L) (Tereos, Moussy-le-Vieux, France), yeast extract (25 g/L) (Procelys by Lesaffre, Maisons-Alfort, France), KH_2_PO_4_ (2 g/L) (Merck, Darmstadt, Germany), CH_3_COONa (6 g/L) (Penta Chemicals, Prague, Czech Republic), MgSO_4_ (0.3 g/L) (Chem-Lab analytical, Zedelgem, Belgium), and MnSO_4_ (0.005 g/L) (Honeywell International, Charlotte, NC, USA) with an initial pH of 6.5. The cultures were incubated at 37 °C for 24 h.

### 2.2. Production of Functional Food Ingredients

#### 2.2.1. Immobilization of *L. paracasei* FBM_1327 on Sunflower Seeds

Cell biomass of 1 L freshly grown *L. paracasei* FBM_1327 (9.32 log cfu/mL) was harvested by centrifugation (8500 rpm, 15 min, 4 °C) and, subsequently, washed with 100 mL sterile ¼ Ringer’s solution (VWR International GmbH, Radnor, PA, USA) and centrifuged again. For the immobilization process, commercial whole hulled confection sunflower seeds (Mr. Grand, Masoutis, Thessaloniki, Greece) (previously pre-heated at 140 °C for 30 min to avoid contamination) were used as a natural immobilization carrier. The harvested cells were resuspended in sterile ¼ Ringer’s solution to the initial culture volume, and sunflower seeds were immersed in cell suspension in a ratio of 80% *w/v* and left undisturbed at room temperature for 4 h. Immobilization was achieved through the natural adhesion of bacterial cells onto the sunflower seed surface. At the end of the immobilization process, the mixture was strained and washed with 100 mL of sterile ¼ Ringer’s solution in order to remove the non-immobilized (free) cells.

#### 2.2.2. Freeze-Drying of Immobilized Cells

Immobilized cells were placed overnight at −80 °C and, subsequently, freeze-drying took place on a BenchTop Pro (Virtis, SP Scientific, Warminster, PA, USA) freeze-dryer under vacuum (30–35 Pa) at condenser temperature −101 °C, for 24 h. Freeze-dried free cells were also produced for comparison reasons, according to Prapa et al. (2023) [9].

### 2.3. Survival of Immobilized L. paracasei FBM_1327 Cells During Simulated Gastric Conditions

The cell viability of immobilized *L. paracasei* FBM_1327 cells on sunflower seeds was assessed during passage through the GI tract, following the method described by Nelios et al. (2023) [10]. Initially, free or immobilized *L. paracasei* FBM_1327 cells were mixed with simulated salivary fluid (SSF) containing α-amylase (75 U/mL) and lysozyme (50 U/mL) and incubated for 2 min. In case of immobilized cells, the culture-SSF solution was homogenized using an iMix bag mixer (Interlab, Mourjou, France) to simulate the oral phase. Subsequently, the samples were separated from the liquid phase and exposed to simulated gastric fluid (SGF) supplemented with 2000 U/mL pepsin from porcine gastric mucosa (≥3200 U/mg) (Sigma-Aldrich, St. Louis, MO, USA) at 37 °C for 110 min. During this stage, the pH was gradually reduced from 4.9 to 3.0 to mimic gastric transition conditions. Then, the samples were strained, and the liquid phase was discarded, followed by exposure to simulated intestinal fluid (SIF) containing 100 U/mL porcine pancreatic mucin (8 *×* USP specifications) (Sigma-Aldrich, St. Louis, MO, USA) and incubation at 37 °C for 120 min. After incubation, the samples were strained, and the liquid phase was discarded. Samples of 1 mL of free cells or 5 g of immobilized cells were collected at the beginning of the procedure and at the end of each simulated phase and cell loads of immobilized *L. paracasei* FBM_1327 cells were determined by 10-fold serial dilutions and plate counting on MRS agar (Condalab, Madrid, Spain) and incubation anaerobically (2.5 L anaerobic jar; Sachets Merck Millipore, Darmstadt, Germany) at 37 °C for 72 h, following the method described by Prapa et al. (2025) [11]. The survival rates were estimated following the equation [10]:

Survival rate (%) = (log cfu a/log cfu b) × 100,

where a refers to cell loads after each simulated digestion phase, and b refers to the cell loads at the beginning of the simulated digestion phase.

### 2.4. Effect of Storage on Cell Viability of Immobilized L. paracasei FBM_1327 Cells

The wet and freeze-dried free and immobilized *L. paracasei* FBM_1327 cells were placed into sterile containers and stored at room temperature (18–22 °C) or 4 °C for 180 days. In all cases, cell loads of *L. paracasei* FBM_1327 cells were recorded at regular intervals.

### 2.5. Functional Cream Cheese Preparation

Cream cheese (“Arla”, Central Denmark Region, Denmark) (skimmed milk and cream, 34% fat, 0.8% salt) purchased from a local market was fortified with freeze-dried immobilized *L. paracasei* FBM_1327 cells on whole sunflower seeds (2 g/10 g of product) (sample CCI), resulting in 8 log cfu/g of cream cheese. For comparison reasons, cream cheese containing freeze-dried free cells (0.025 g/10 g of product) (sample CCF), resulting in 8 log cfu/g of cream cheese, was also prepared. Additionally, a commercial product with no *L. paracasei* FBM_1327 cells (sample CCC) was used as a control. All products were stored at refrigerated temperature (4 °C) for 28 days to monitor the shelf life of the products.

### 2.6. Microbiological Analyses

#### 2.6.1. Determination of *L. paracasei* FBM_1327 Cell Loads

For the determination of cell levels of wet and freeze-dried free or immobilized *L. paracasei* FBM_1327 cells and their survival rate, the procedure described in Section 2.3 was followed.

In order to determine *L. paracasei* FBM_1327 counts in cream cheese products, 10 g of samples were homogenized with 90 mL of sterile 0,1% *w/v* Buffered Peptone water (Lab M, Heywood, UK), followed by 10-fold serial dilutions in ¼ Ringer’s solution and plate counting on MRS agar [11].

#### 2.6.2. Monitoring of Microbial Contamination

Possible presence of food spoilage or pathogenic microorganisms during storage was checked in accordance with European Commission Regulation (EC) No 2073/2005 [24] by determining:

• total mesophilic counts on Plate Count Agar (PCA) (Condalab) at 30 °C for 72 h;
• lactococci counts on M17 agar (PCA) (Condalab) at 30 °C for 72 h;
• staphylococci counts in Baird Parker agar (Condalab) enriched with egg yolk tellurite (Condalab) at 37 °C for 48 h;
• *Listeria monocytogenes* on *Listeria* agar base Palcam ISO (Condalab) supplemented with Palcam *Listeria* Selective Supplement (Condalab) at 37 °C for 48 h;
• *Salmonella* spp. on Xylose Lysine Deoxycholate (X.L.D.) Agar (Condalab) at 37 °C for 48 h;
• coliforms on Violet Red Bile agar (Condalab) at 30 °C for 24 h;
• *Enterobacteriacae* on Violet Red Bile Glucose agar at 37 °C for 24 h, and
• yeasts/molds counts in Malt agar (Condalab) at 30 °C for 72 h.

### 2.7. Physicochemical Analyses

Water activity (a_w_), pH, and titratable acidity were determined according to Pavlatou et al. (2023) [12].

### 2.8. Minor Volatiles Content of Functional Cream Cheese

Samples of each cream cheese product (20 g) were collected at the beginning and after 14 days of storage and analyzed for their minor volatile profile using the HS-SPME GC/MS technique [6890N GC, 5973NetworkedMS MSD (Agilent Technologies, Santa Clara, CA, USA)], as previously described [11].

### 2.9. Sensory Evaluation of Functional Cream Cheese Products

The effect of free or immobilized *L. paracasei* FBM_1327 cells on sensory attributes of functional cream cheese products was assessed in accordance with ISO 6658:2017 [25] and ISO 8589:2007 [26], as described by Pavlatou et al. (2023) [12]. A commercial cream cheese product was used as a control. The samples were evaluated by 15 untrained panelists, regular consumers of dairy products, providing scores on a 0–5 scale (0: unacceptable, 5: exceptional), regarding the aroma, texture, taste, and overall quality. The samples were coded and presented in random order.

### 2.10. Experimental Design and Statistical Analysis

All experiments were performed at least in duplicate (two independent replicates). Statistical analysis was carried out using Analysis of Variance (ANOVA) through Statistica (v. 10.0, StatSoft, Tulsa, OK, USA). Significant differences (*p* < 0.05) were determined with Duncan’s test.

## 3. Results and Discussion

### 3.1. Evaluation of Cell Survival During In Vitro Digestion Model

**Figure 1:**
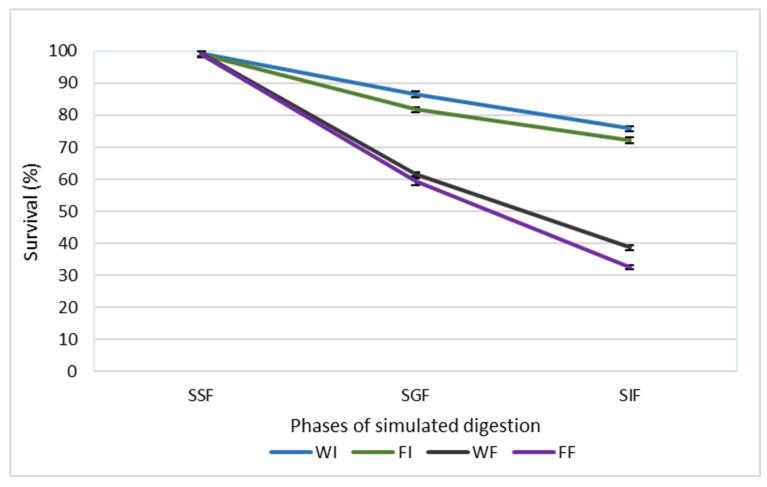
Survival rates (%) of free and immobilized *L. paracasei* FBM_1327 cells during *in vitro* digestion. The values are expressed as mean values ± standard deviation. SSF, simulated salivary fluid; SGF, simulated gastric fluid; SIF, simulated intestinal fluid; WI, wet immobilized cells on sunflower seeds; FI, freeze-dried immobilized cells; WF, wet free cells; FF, freeze-dried free cells.

The potential protective effect of cell immobilization on the viability of *L. paracasei* FBM_1327 cells was evaluated by performing a static in vitro digestion protocol, and the results are presented in Figure 1. Survival rates were significantly (*p* < 0.05) affected by both the state (wet or freeze-dried) and the nature (free or immobilized) of the cultures. More specifically, at the end of the *in vitro* digestion protocol, higher (*p* < 0.05) survival rates were observed by immobilized compared to the free cells, while freeze-dried cultures exhibited lower (*p* < 0.05) survival rates than wet cultures, a result that could be attributed to the stress during freeze-drying [27]. Incubation in the simulated salivary fluid had no effect (*p* > 0.05) on cell viability, and similar survival rates (*p* > 0.05) in all cases were recorded. At the end of the simulated intestinal phase, wet and freeze-dried immobilized cells exhibited survival rates of 75.82% and 72.24%, respectively. Survival rates up το 38.68 and 32.44% were observed for wet and freeze-dried free cultures, respectively.

The digestive process poses a significant challenge to microbial viability due to the presence of harsh conditions, including low pH, bile salts, and digestive enzymes. Cell immobilization on natural food carriers has been proposed as a strategy to overcome this bottleneck, due to the protective effect that has been previously reported in similar studies [10,28,29,30]. The entrapment or attachment of cells in a food matrix provides a protective effect on cells, acting as a barrier to harsh conditions prevailing in the GI tract [31,32]. Furthermore, it has been reported that food carriers may increase the pH values in the gastric phase, behaving like a buffer [31]. Indeed, Sidira et al. (2010) [28] mentioned that higher cell loads were recorded by immobilized *L. casei* ATCC 393 on apple pieces, while free cells were more susceptible to gastric and intestinal conditions. Likewise, Nelios et al. (2023) [10] reported survival rates > 51% after in vitro digestion in the case of immobilized cultures, while free cells exhibited significantly lower survivability (36.29%). Notably, Faye et al. (2012) [33] mentioned that the strain *L. paracasei* INF456, isolated from cheese, showed higher survival rates after in vitro digestion, where fermented milk was used as a carrier matrix, compared to free cells [33].

### 3.2. Storage of Immobilized L. paracasei FBM_1327 Cells

#### 3.2.1. Cell Loads of Immobilized *L. paracasei* FBM_1327 Cells During Storage

For probiotic-enriched functional foods, maintaining high cell loads during both processing and storage remains a key challenge for the food industry. Storage temperature significantly affects cell viability, with storage at 4–5 °C considered optimal for the maintenance of high cell levels [34,35]. The requirement for refrigerated storage increases product maintenance costs, whereas dried products are considered more cost-effective and easier to transport, due to higher stability and the ability to be stored at room temperature [9]. In this vein, freeze-dried immobilized cells on sunflower seeds were stored at room temperature and 4 °C for up to 180 days, and cell viability was monitored at various intervals. Simultaneously, cell loads of freeze-dried free cells were recorded for comparison purposes. The results are presented in Table 1.

Cell immobilization contributed to the survival of the cultures at higher levels (*p* < 0.05) compared to free cells, in all cases. Cell viability was affected (*p* < 0.05) by all factors [temperature, storage time, and culture form (wet or freeze- dried, free or immobilized)], and a strong interaction (*p* < 0.05) was observed between the factors. As expected, immobilization and low storage temperatures had a significant (*p* < 0.05) effect on cell survival, while freeze-drying contributed positively to maintaining the levels at a concentration ≥7 log cfu/g for a longer time period.

During storage at room temperature, significantly (*p* < 0.05) higher survival rates (89.23 ± 0.14%) in freeze-dried immobilized cells compared to freeze-dried free cells (74.33 ± 0.11%) were noticed after 30 days of storage, while the corresponding values for wet free cells and wet immobilized cells were 45.50 ± 0.15% and 50.09 ± 0.08%. As the storage time was extending, cell loads were recorded <1 log cfu/g in wet and freeze-dried free cells, and in wet immobilized cells. In the case of freeze-dried immobilized cells, 6.55 ± 0.03 log cfu/g were recorded after 90 days of storage.

On the other hand, storage at low temperatures led to maintenance of high cell loads for longer (*p* < 0.05) periods compared to storage at room temperature, in all cases examined. Indeed, freeze-dried immobilized cell levels were maintained at >8.40 log cfu/g after 3 months of storage, while on the 180th day, the cell loads remained higher than the minimum recommended concentration for conferring health effects (7.55 ± 0.03 log cfu/g). Meanwhile, freeze-dried free cells exhibited lower (*p* < 0.05) survival rates throughout storage compared to freeze-dried immobilized cultures. More specifically, cell viability of freeze-dried free cultures dropped significantly (*p* < 0.05) to 7.87 ± 0.09 log cfu/g after 90 days of storage, while their cell loads were detected <1 log cfu/g after 6 months. In case of wet cultures, immobilized cells exhibited 50.40 ± 0.24% survival rates after 90 days of storage, while the cell viability of free cells was recorded as <1 log cfu/g.

According to the literature, freeze-drying contributes to maintaining high cell viability during storage of probiotic strains, consistent with our results [9,13]. Several studies supported that low storage temperatures prolong cell survival, compared to higher temperatures [34,35,36]. During storage, saturated fatty acids in the cell membrane increase due to lipid oxidation. Lipid oxidation, which is favored at high storage temperatures, is accompanied by the formation of free radicals and, subsequently, damage to DNA and the cell membrane [37,38]. At the same time, low storage temperatures (10 °C) are recommended for the long-term preservation of the quality characteristics of food ingredients, as it is estimated that low temperatures prevent lipid oxidation and thus limit deterioration [39]. Cell immobilization has been proposed as an effective approach to ensure maintenance of high cell viability during storage, providing a protective microenvironment by reducing the impact of environmental stress factors [8]. Indeed, immobilized *L. paracasei* FMB_1327 cells exhibited higher survival rates compared to free cells. Specifically, a sharp reduction in cell counts of freeze-dried free cells was recorded after prolonged storage. This decline may be associated with the accumulation of irreversible damage to the cell wall, cell membrane, and DNA occurring during storage [40,41].

#### 3.2.2. Water Activity (a_w_) Values of Immobilized *L. paracasei* FBM_1327 Cells During Storage

Water activity (a_w_) was also monitored during storage (Table 2). Water activity is considered a critical factor, which is partially responsible for the safety of the product during storage [42]. For long-term storage of dried cells, water activity values are recommended to range < 0.25 [37]. A_w_ values of freeze-dried immobilized cells were recorded at low levels after freeze-drying (0.083 ± 0.05), as suggested for dry food ingredients [43], while the initial values of wet immobilized cells were 0.954 ± 0.02. A_w_ was affected (*p* < 0.05) by temperature, storage time, and culture form (wet or freeze-dried, free or immobilized), while a strong interaction (*p* < 0.05) was observed between the factors. In particular, at room temperature, an increase (*p* < 0.05) in a_w_ values was observed in freeze-dried cultures [44]. Of note, a decrease in a_w_ in wet cultures was associated with reduced cell loads. The a_w_ of a product is considered an important factor for the survival of beneficial microorganisms, but also for the growth of spoilage microbes [42,45]. It has been reported that under certain conditions, where a_w_ and moisture are increased, water may act as a plasticizer and increase molecular mobility, resulting in carbohydrate crystallization and loss of cell viability. The relationship between a_w_ and cell survival is a complex phenomenon and is likely influenced by the initial water content and the tendency to equilibrate with the storage environment [45]. More specifically, Jiménez et al. (2015) evaluated the stability of encapsulated *L. paracasei* subsp. *paracasei* LBC81 strainstored at several water activities and temperatures, reporting a decrease in viability of the strain at a_w_ ≥ 0.5 [46]. According to this study, reduced viability during storage may be associated with changes in water mobility at specific a_w_ levels, leading to osmotic stress, membrane structural alterations, and increased permeability. Moreover, dehydration–rehydration phenomena can induce lipid phase transitions and protein destabilization, resulting in irreversible cellular damage.

### 3.3. Production of Functional Cream Cheese

Cream cheese is an unripened cheese with a soft, spreadable texture, which could act as a vehicle of beneficial microorganisms, due to its favorable pH values, fat content, solid matrix, and storage conditions [19]. In this vein, freeze-dried immobilized *L. paracasei* FBM_1327 cells on sunflower seeds were incorporated as a functional food ingredient into cream cheese (CCI sample), and cell viability of *L. paracasei* FBM_1327, as well as physicochemical parameters, minor volatiles profile, and sensory characteristics of the functional cream cheese were evaluated. For comparison reasons, cream cheese fortified with freeze-dried free *L. paracasei* FBM_1327 cells (CCF sample) was produced, while cream cheese without *L. paracasei* FBM_1327 cells were used as a control (CCC sample) (Figure S1).

#### 3.3.1. Viability of *L. paracasei* FBM_1327 Cells in Functional Cream Cheese

The cell loads of *L. paracasei* FBM_1327 cells during storage are presented in Table 3. The initial cell levels in CCF and CCI samples recorded were 8.01 ± 0.07 and 8.21 ± 0.01 log cfu/g, respectively. According to the results, no significant (*p* > 0.05) differences were observed in cell viability of immobilized cells in the CCI sample until the 14th day of storage, while at the end of the storage time, cell levels were 8.11 ± 0.01 log cfu/g. In case of the CCF sample, cell viability was significantly (*p* < 0.05) decreased during storage (7.88 ± 0.07 and 7.75 ± 0.14 after 7 and 14 days, respectively). At the end of the storage period, free cells were detected in the CCF sample at 7.51 ± 0.11 log cfu/g. Noticeably, no growth (<1 log cfu/g) of spoilage or pathogenic microorganisms was observed. Furthermore, it should be noted that lactic acid bacteria (LAB) were below the detection limit in the control product (CCC), indicating that it is highly unlikely that the lactobacilli counts reported in the case of the fortified samples (CCF and CCI) were attributed to the indigenous LAB rather than *L. paracasei* FBM_1327 cells. In general, immobilized cells maintained higher (*p* < 0.05) cell loads than free cells, indicating the positive effect of cell immobilization, as well as the favorable compositional characteristics of sunflower seeds, mainly due to their high fatty acids and fiber content [47].

Ensuring high cell concentration of probiotics in foods until the time of consumption is a significant challenge for the food industry. Both free and immobilized cells were detected in higher cell concentrations compared to the minimum recommended values (>7 log cfu/g) in accordance with previous studies supporting that cream cheese could act as an excellent probiotic vehicle [18,20,48]. For instance, Ningtyas et al. (2019) [20] mentioned that cell levels of *L. rhamnosus* strain were maintained above 10^6^ cfu/g during storage of cream cheese products for 35 days. In the same manner, Santini et al. (2012) [48] reported that the strain *L. paracasei* Lpc-37 remained in loads >7.5 log cfu/g during storage for 21 days.

#### 3.3.2. pH and Titratable Acidity (TA) Values in Functional Cream Cheese

The pH and TA values are considered important physicochemical parameters for evaluating both cell viability and overall product quality. Changes in these parameters may affect product texture and flavor, while they may reflect ongoing metabolic activity within the product [20,49,50]. Therefore, their values were also monitored during storage, and the results are presented in Table 4. In all samples (CCC, CCF, and CCI), pH values ranged from 4.94 to 4.98 after preparation. During storage, no significant (*p* > 0.05) differences in pH values of the CCC sample were observed, while in products enriched with *L. paracasei* FBM_1327, a decrease (*p* < 0.05) in pH after 14 days of storage was noticed (4.78 ± 0.01 and 4.81 ± 0.04 for CCF and CCI samples, respectively). Regarding TA, the initial values ranged from 0.35% to 0.38%. In samples CCF and CCI, an increase (*p* < 0.05) in TA was observed, reaching values of 0.67 ± 0.02% and 0.70 ± 0.02%, respectively, after 28 days of storage. The fortification of cream cheese with *L. paracasei* FBM_1327 cells led to higher (*p* < 0.05) TA values and, consequently, lower (*p* < 0.05) pH compared to the control (CCC) sample in accordance with previous studies, probably attributed to the production of lactic acid and other organic acids as a result of the metabolic activity of *L. paracasei* FBM_1327 cells [20,49,51].

#### 3.3.3. Minor Volatiles Profile of Functional Cream Cheese

**Figure 2:**
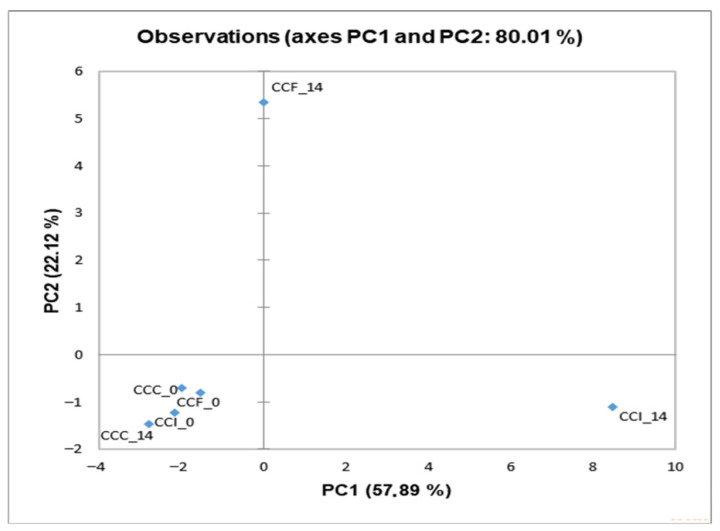
Principal Component Analysis (PCA) plot of minor volatiles detected in functional cream cheese samples containing free or immobilized *L. paracasei* FBM_1327 cells on sunflower seeds. CCC, control cream cheese; CCF, cream cheese with free *L. paracasei* FBM_1327 cells; CCI, cream cheese with immobilized *L. paracasei* FBM_1327 cells on sunflower seeds. The storage duration (days) is shown at the end of the sample codes.

The characteristic flavor of a product is a result of the combination of various volatile compounds, such as ketones, aldehydes, acids, and esters [52]. Several studies have reported that the fortification with probiotics may influence the minor volatile profile of products, influencing their sensory attributes [53,54]. As so, the volatile compounds of the cream cheese products were determined by HS-SPME GC/MS analysis (Table S1), and PCA was applied to the results (Figure 2). No significant differences were detected after preparation (day 0), as all samples were gathered at the bottom left quadrant of the plot. The enrichment with free or immobilized *L. paracasei* FBM_1327 cells contributed to the products’ volatile profile during storage, and a concentration increase (*p* < 0.05) was observed for most compounds (after 14 days), thus affecting the products’ sensory characteristics (Table 5). Notably, supplementation with LAB is often sought for imparting unique flavor attributes to dairy products [12,49,55]. As expected, the most abundant compound (in all samples) was acetic acid, a main byproduct of lactic acid bacteria fermentation and primarily linked to vinegar-like taste.

**Figure 3:**
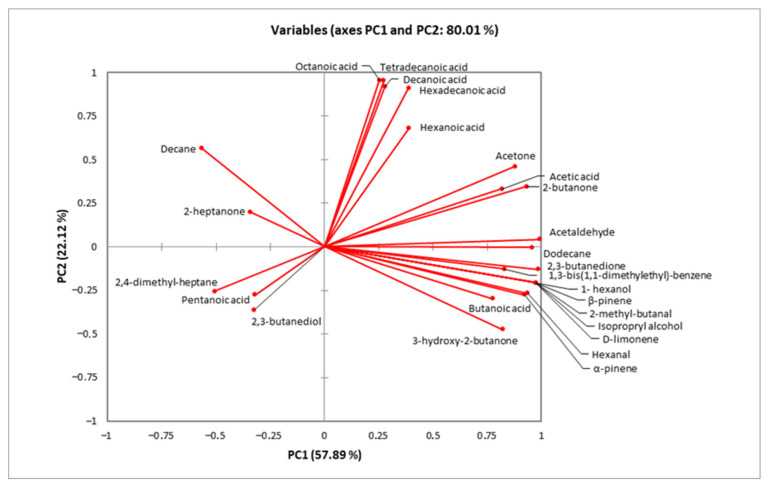
Minor volatile compounds determined by HS-SPME GC/MS analysis in functional cream cheese samples.

Regarding samples containing *L. paracasei* FBM_1327 cells, CCF was located at the top of the diagram (Figure 2) and was mostly associated with organic acids positively correlating to PC2 (Figure 3). Increased organic acid concentrations have been previously attributed to proteolytic and lipolytic activity of LAB [23,56,57]. Thus, the higher (*p* < 0.05) concentrations detected after 14 days can be related to the metabolic activity of *L. paracasei* FBM_1327 cells and may also contribute to the product’s quality. Specifically, hexanoic acid is mostly known for its pungent odor, octanoic acid is responsible for its “goaty” and waxy flavor, while decanoic acid is usually related to rancid and fatty characteristics [52,58].

On the other hand, the CCI sample was located in the lower right side of the plot (Figure 2) and was mostly affected by carbonyls, alcohols, and other compounds (Figure 3). Ketones are important components in cheese, as they contribute to the cheese flavor due to their low threshold [52,59]. Specifically, acetoin (3-hydroxy-2-butanone) and diacetyl (2,3-butanedione) are associated with creamy and buttery flavors and are considered significant contributors for the positive consumer perception for dairy products. Their concentrations were significantly (*p* < 0.05) higher when *L. paracasei* FBM_1327 cells were used, thus indicating the microorganism’s potential use for determining the product’s distinct flavor [20]. The presence of acetaldehyde is often considered typical for dairy products containing *Lactobacillus* spp. [56,60]. It was detected in increased levels in products containing *L. paracasei* FBM_1327 cells (after 14 days of storage) and is associated with green apple and nutty notes [61,62]. Meanwhile, the presence of 1- hexanol (detected only in the CCI sample) could be attributed to sunflower seeds and is considered to have a positive impact, offering nutty and fruity aroma notes to the cream cheese [63].

The rest of the detected compounds included butanoic acid, 2,3-butanediol, 2-butanone, d-limonene, a-pinene, b-pinene, 2-heptanone, 2,4-dimethyl-heptane, isopropyl alcohol, acetone, hexanal, pentanoic acid, 2-methyl-butanal, decane, tetradecanoic acid, 1,3-bis(1,1-dimethylethyl)-benzene, hexadecanoic acid, and dodecane at concentrations ranging from 0.01 to 1.02 mg/kg. Of note, the presence of terpenes (mainly a-pinene, b-pinene, and hexanal) in the CCI sample could be attributed to the use of sunflower seeds as immobilization carrier [64,65].

#### 3.3.4. Sensory Evaluation

The sensory attributes of a product significantly affect the consumers’ acceptability. For this reason, the functional cream cheese samples were evaluated for their sensory properties, and the results are summarized in Table 5. The evaluated cream cheese samples exhibited the typical characteristics expected for this product, including a mild milk-like aroma, creamy and buttery texture, and a balanced sweet-sour and salted taste profile [66]. No significant differences were observed between CCC and CCF samples in terms of overall quality (3.56 ± 0.73 and 3.53 ± 0.85, respectively), indicating that the incorporation of free *L. paracasei* FBM_1327 cells did not affect the product’s acceptability. Although significantly higher (*p* < 0.05) salted taste scores were recorded in samples containing the presumptive probiotic strain, these differences remained within acceptable limits and did not adversely affect overall sensory perception. Interestingly, the sample containing immobilized cells (CCI) received the highest (*p* < 0.05) overall quality score (3.78 ± 0.52). This result may be associated with the volatile profile observed in the CCI sample, where higher concentrations of acetoin and diacetyl compounds linked to creamy and buttery flavor notes were detected (Table 5). Similarly, Tologana et al. (2022) [67] reported that the presence of high levels of acetoin and diacetyl in cream cheese fortified with *L. plantarum* strain is linked with the high score obtained regarding aroma and taste [67]. Moreover, the presence of terpenes may have contributed subtle aromatic nuances, enhancing consumer perception. Overall, the results indicated that fortification of cream cheese with *L. paracasei* FBM_1327 cells did not compromise sensory quality and may contribute positively to flavor complexity.

## 4. Conclusions

Freeze-dried immobilized *L. paracasei* FBM_1327 cells (previously evaluated for their probiotic potential) on sunflower seeds were evaluated as a stable food ingredient regarding their ability to survive in an *in vitro* digestion model, as well as during storage at ambient and refrigerated temperatures in comparison to free cells. Higher survival rates were recorded in immobilized cells during simulated digestion conditions compared to free cells, while freeze-dried immobilized cells exhibited cell loads above the minimum recommended concentration (>7.5 log cfu/g) for conferring a health-promoting effect throughout storage at 4 °C for up to 6 months. Subsequently, freeze-dried free or immobilized cells were used for the enrichment of cream cheese, and cell loads >8 log cfu/g were initially achieved. Cell viability of the immobilized cells remained stable in functional cream cheese during storage, maintaining levels above the minimum recommended concentration for probiotic efficacy. At the same time, their incorporation contributed to the enhancement of the volatile profile, particularly increasing the content of compounds associated with creamy and buttery notes, which was reflected in slightly improved overall sensory scores. Importantly, no negative effects on product acceptability were observed, supporting the potential of the immobilized cells as an adjunct functional food ingredient.

In short, our findings supported the potential of freeze-dried immobilized *L. paracasei* FBM_1327 cells on sunflower seeds to serve as a stable food ingredient for the development of functional dairy products, while the proposed immobilization method could be considered suitable for scale-up and potential industrial application. However, further research is required to validate probiotic efficacy and safety through randomized controlled clinical trials, focusing on GI health parameters and modulation of the gut microbiome.

## Figures and Tables

**Table 1:** Effect of freeze-drying and storage at room (18–22 °C) and refrigerated (4 °C) temperatures on cell loads (log cfu/g) and survival rates (%) of immobilized *L. paracasei* FBM_1327 cells on sunflower seeds compared to free cells.

Storage Time (Days)	Wet Immobilized Cells		Wet Free Cells		Freeze-Dried Immobilized Cells		Freeze-Dried Free Cells	
	18–22 °C	4 °C	18–22 °C	4 °C	18–22 °C	4 °C	18–22 °C	4 °C
	llog cfu/g							
0	8.88 ± 0.05 ^a^	8.75 ± 0.11 ^a^	11.74 ± 0.04	11.74 ± 0.04	9.01 ± 0.11 ^a^	9.01 ± 0.11 ^a^	10.75 ± 0.01	10.75 ± 0.01
30	4.04 ± 0.02 ^a,b^	7.88 ± 0.09 ^a,b^	5.88 ± 0.02 ^b^	7.74 ± 0.04 ^b^	8.04 ± 0.09 ^a,b^	8.77 ± 0.09 ^a,b^	7.99 ± 0.08 ^b^	9.68 ± 0.05 ^b^
90	0	6.22 ± 0.03 ^a,b,c^	0	3.85 ± 0.06 ^b,c^	6.55 ± 0.03 ^a,b,c^	8.41 ± 0.03 ^a,b,c^	0	7.87 ± 0.09 ^b,c^
180	0	0	0	0	0	7.55 ± 0.03 ^a,b,c,d^	0	0
	Survival rate (%)							
30	45.50 ± 0.15 ^a,b^	90.06 ± 0.22 ^a,b^	50.09 ± 0.08 ^b^	65.93 ± 0.12 ^b^	89.23 ± 0.14 ^a,b^	97.34 ± 0.15 ^a,b^	74.33 ± 0.11 ^b^	90.05 ± 0.21 ^b^
90	N.D.	71.09 ± 0.11 ^a,b,c,^	N.D.	32.79 ± 0.11 ^b,c^	72.70 ± 0.05 ^a,b,c^	93.34 ± 0.09 ^a,b,c^	N.D.	73.21 ± 0.18 ^b,c^
180	N.D.	N.D.	N.D.	N.D.	0	83.80 ± 0.17 ^a,b,c,d^	N.D.	N.D.

The values are expressed as means ± standard deviation. Different superscript letters indicate significant differences: ^a^ *p* < 0.05 vs. free cells; ^b^ *p* < 0.05 vs. day 0; ^c^ *p* < 0.05 vs. day 30; ^d^ *p* < 0.05 vs. day 90.

**Table 2:** Effect of freeze-drying and storage at room (18–22 °C) and refrigerated (4 °C) temperatures on water activity of immobilized *L. paracasei* FBM_1327 cells on sunflower seeds compared to free cells.

Storage Time (Days)	Wet Immobilized Cells		Wet Free Cells		Freeze-Dried Immobilized Cells		Freeze-Dried Free Cells	
	18–22 °C	4 °C	18–22 °C	4 °C	18–22 °C	4 °C	18–22 °C	4 °C
0	0.954 ± 0.02	0.954 ± 0.02	0.915 ± 0.02	0.915 ± 0.02	0.083 ± 0.05	0.083 ± 0.05	0.072 ± 0.03	0.072 ± 0.03
30	0.912 ± 0.01 ^a^	0.932 ± 0.03 ^a^	0.895 ± 0.01 ^a^	0.898 ± 0.01 ^a^	0.097 ± 0.03 ^a^	0.091 ± 0.01 ^a^	0.095 ± 0.01 ^a^	0.091 ± 0.02 ^a^
90	N.D.	0.925 ± 0.01 ^a,b^	N.D.	0.891 ± 0.02 ^a,b^	0.121 ± 0.01 ^a,b^	0.097 ± 0.05 ^a,b^	N.D.	0.107 ± 0.01 ^a,b^
180	N.D.	N.D.	N.D.	N.D.	N.D.	0.121 ± 0.03 ^a,b,c^	N.D.	N.D.

The values are expressed as means ± standard deviation. Different superscript letters indicate significant differences: ^a^ *p* < 0.05 vs. day 0; ^b^ *p* < 0.05 vs. day 30; ^c^ *p* < 0.05 vs. day 90.

**Table 3:** Levels (log cfu/g) of lactobacilli in functional cream cheese during storage.

Days of Storage	Sample		
	CCC	CCF	CCI
0	<1	8.01 ± 0.07 ^h^	8.21 ± 0.01 ^a,b^
3	<1	7.79 ± 0.05 ^c^	8.19 ± 0.02 ^b^
7	<1	7.69 ± 0.07 ^c^	8.15 ± 0.04 ^a,b^
14	<1	7.49 ± 0.14 ^g^	8.12 ± 0.1 ^a,d^
21	<1	7.37 ± 0.23 ^f^	8.09 ± 0.15 ^d^
28	<1	7.21 ± 0.11 ^e^	8.02 ± 0.01 ^i^

The levels are expressed as mean values ± standard deviation. Significant differences (*p* < 0.05) are denoted by different letters in superscript. CCC: control cream cheese; CCF: cream cheese with free *L. paracasei* FBM_1327 cells; CCI: cream cheese with immobilized *L. paracasei* FBM_1327 cells on sunflower seeds.

**Table 4:** Effect of *L. paracasei* FBM_1327 on pH and TA (%) values of functional cream cheese.

Parameter	Storage Time (Days)	CCC	CCF	CCI
pH	0	4.98 ± 0.06	4.96 ± 0.05	4.94 ± 0.01
	3	4.99 ± 0.02	4.94 ± 0.01	4.95 ± 0.02
	7	5.01 ± 0.08	4.91 ± 0.02 ^a,b^	4.90 ± 0.02 ^a,b^
	14	4.97 ± 0.05	4.85 ± 0.04 ^a,b^	4.88 ± 0.03 ^a,b^
	21	4.99 ± 0.02	4.82 ± 0.02 ^a,b^	4.85 ± 0.01 ^a,b^
	28	4.98 ± 0.03	4.78 ± 0.01 ^a,b^	4.81 ± 0.04 ^a,b^
TA (%)	0	0.38 ± 0.04	0.37 ± 0.02	0.35 ± 0.02
	3	0.40 ± 0.02	0.41 ± 0.04 ^b^	0.37 ± 0.04
	7	0.42 ± 0.05 ^b^	0.45 ± 0.02 ^b^	0.43 ± 0.02 ^b^
	14	0.45 ± 0.03 ^b^	0.57 ± 0.03 ^a,b^	0.59 ± 0.06 ^a,b^
	21	0.40 ± 0.03	0.61 ± 0.05 ^a,b^	0.65 ± 0.03 ^a,b^
	28	0.38 ± 0.02	0.67 ± 0.02 ^a,b^	0.70 ± 0.02 ^a,b^

The levels are expressed as mean values ± standard deviation. Different superscript letters indicate significant differences: ^a^ *p* < 0.05 vs. CCC; ^b^ *p* < 0.05 vs. day 0. CCC, control cream cheese; CCF, cream cheese with free *L. paracasei* FBM_1327 cells; CCI, cream cheese with immobilized *L. paracasei* FBM_1327 cells on sunflower seeds.

**Table 5:** Sensory evaluation of functional cream cheeses.

Sensory Attributes	Description	CCC	CCF	CCI
Aroma	Milk-like	3.13 ± 0.11	3.10 ± 0.22	3.15 ± 0.12
Texture	Creamy	3.56 ± 0.31	3.45 ± 0.27	3.55 ± 0.11
	Buttery	3.21 ± 0.14	3.18 ± 0.33	3.27 ± 0.18
Taste	Salted	1.75 ± 0.35	2.03 ± 0.21	2.35 ± 0.54
	Sweet-sour	2.55 ± 0.40	2.25 ± 0.11	2.11 ± 0.31
Overall quality		3.56 ± 0.73	3.53 ± 0.85	3.78 ± 0.52

The levels are expressed as mean values ± standard deviation. CCC, control cream cheese; CCF, cream cheese with free *L. paracasei* FBM_1327 cells; CCI, cream cheese with immobilized *L. paracasei* FBM_1327 cells on sunflower seeds.

## Data Availability

The original contributions presented in this study are included in the article/Supplementary Material. Further inquiries can be directed to the corresponding author.

## Supplementary Materials

The following supporting information can be downloaded at: https://www.mdpi.com/article/10.3390/microorganisms14030671/s1, Figure S1: (A) Commercial cream cheese sample (CCC). (B) Cream cheese sample fortified with immobilized *L*. *paracasei* FBM_1327 cells on sunflower seeds (CCI). Table S1: Minor volatiles (mg/kg) detected by HS-SPME GC/MS analysis in cream cheese products fortified with freeze-dried free or immobilized *L*. *paracasei* FBM_1327 cells on sunflower seeds at day 0 and after 14 days of storage in comparison to a commercially available product.
