# Supplementary material for: Immobilized Lacticaseibacillus paracasei on Sunflower Seeds as a Stable Functional Ingredient for Cream Cheese
Source: Microorganisms. 2026 Mar 16;14(3):671. doi: 10.3390/microorganisms14030671 (PMC13029329; doi:10.3390/microorganisms14030671)
Supplement: Supplementary file 1 [file microorganisms-14-00671-s001.zip › microorganisms-4119084-supplementary.pdf]

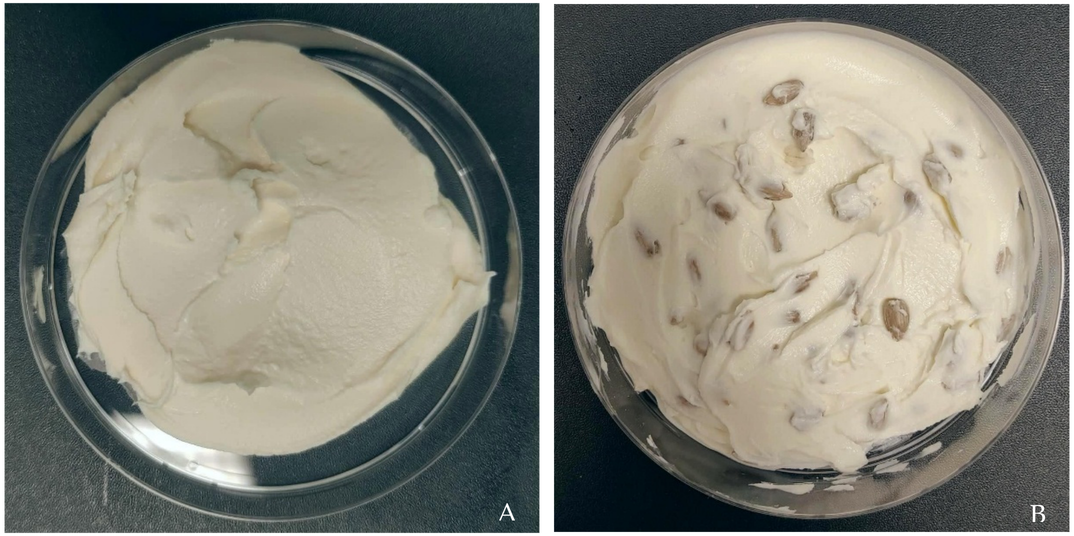

Figure S1. A) Commercial cream cheese sample (CCC). B) Cream cheese sample fortified with immobilized *L. paracasei* FBM_1327 cells on sunflower seeds (*CCI*).

Table S1. Minor volatiles (mg/ kg) detected by HS-SPME GC/MS analysis in cream cheese products fortified with freeze-dried free or immobilized *L. paracasei* FBM_1327 cells on sunflower seeds at day 0 and after 14 days of storage in comparison to a commercially available product.

**Compound**	**CCC**		**CCF**		**CCI**	
	**d0**	**d14**	**d0**	**d14**	**d0**	**d14**
2-butanone	n.d.	n.d.	n.d.	< 0.1	n.d.	< 0.1
2-heptanone	< 0.1	0.1 ± 0.1	< 0.1	< 0.1	< 0.1	< 0.1
Acetone	n.d.	n.d.	n.d.	< 0.1	n.d.	< 0.1
2,3-butanedione	0.2 ± 0.1	< 0.1	< 0.1	0.1 ± 0.1	< 0.1	0.4 ± 0.1
3-hydroxy-2-butanone	0.5 ± 0.1	0.6 ± 0.1	0.6 ± 0.2	0.4 ± 0.1	0.4 ± 0.1	0.9 ± 0.1
Acetaldehyde	n.d.	n.d.	n.d.	< 0.1	n.d.	< 0.1
Hexanal	n.d.	n.d.	n.d.	n.d.	< 0.1	< 0.1
2-methyl-butanal	n.d.	n.d.	n.d.	n.d.	n.d.	0.2 ± 0.1
2,3-butanediol	n.d.	n.d.	n.d.	n.d.	n.d.	< 0.1
1-hexanol	n.d.	n.d.	n.d.	n.d.	n.d.	< 0.1
Isopropyl alcohol	n.d.	n.d.	n.d.	n.d.	n.d.	0.2 ± 0.1
Butanoic acid	< 0.1	<0.1	n.d.	n.d.	n.d.	0.1 ± 0.1
Decanoic acid	< 0.1	<0.1	< 0.1	0.2 ± 0.1	n.d.	< 0.1
Tetradecanoic acid	n.d.	n.d.	n.d.	< 0.1	n.d.	< 0.1
Hexanoic acid	0.3 ± 0.1	0.1 ± 0.1	0.1 ± 0.1	0.3 ± 0.1	< 0.1	0.2 ± 0.1
Hexadecanoic acid	n.d.	n.d.	n.d.	0.1 ± 0.1	n.d.	< 0.1
Octanoic acid	0.2 ± 0.1	0.2 ± 0.1	0.2 ± 0.1	0.5 ± 0.1	0.2 ± 0.1	0.3 ± 0.1
Pentanoic acid	n.d.	0.3 ± 0.1	n.d.	n.d.	n.d.	n.d.
Acetic acid	0.6 ± 0.1	0.7 ± 0.3	0.5 ± 0.1	0.8 ± 0.1	0.4 ± 0.1	1.0 ± 0.3
2,4-dimethyl-heptane	n.d.	n.d.	< 0.1	< 0.1	< 0.1	< 0.1
Decane	< 0.1	< 0.1	< 0.1	< 0.1	0.1 ± 0.1	n.d.
Dodecane	n.d.	n.d.	< 0.1	< 0.1	< 0.1	0.2 ± 0.1
1,3-bis(1,1-dimethylethyl)-benzene	< 0.1	< 0.1	0.1 ± 0.1	< 0.1	< 0.1	0.2 ± 0.1
D-limonene	n.d.	n.d.	n.d.	n.d.	n.d.	< 0.1
α -pinene	n.d.	n.d.	n.d.	n.d.	< 0.1	0.2 ± 0.1

β -pinene	n.d.	n.d.	n.d.	n.d.	n.d.	< 0.1

CCC: control cream cheese; CCF: cream cheese with free *L. paracasei* FBM_1327 cells; CCI: cream cheese with immobilized *L. paracasei* FBM_1327 cells on sunflower seeds. Compounds were identified based on comparison of retention times to those of certified standards, Kovats' Retention Indexes, and mass spectra libraries.
